# Correlates of Mild Behavioral Impairment in Older Adults: Protocol for a Scoping Review

**DOI:** 10.2196/60009

**Published:** 2024-07-29

**Authors:** Seolah Yoon, Innhee Jeong, Jennifer Ivy Kim, Dahye Hong, Bada Kang

**Affiliations:** 1 College of Nursing and Brain Korea 21 FOUR Project Yonsei University Seoul Republic of Korea; 2 Mo-Im Kim Nursing Research Institute Yonsei University College of Nursing Seoul Republic of Korea; 3 Department of Nursing Graduate School of Yonsei University Seoul Republic of Korea; 4 Navy Headquarters Republic of Korea Gyeryong Republic of Korea

**Keywords:** mild behavioral impairment, older adults, mild cognitive impairment, subjective cognitive decline, behavioral symptoms, scoping review, protocol, elderly, behavioral impairment, cognitive, cognitive decline, scoping review protocol, older adult, neuropsychological, impairment, behavioral, behavior

## Abstract

**Background:**

Understanding mild behavioral impairment, a relatively recent notion in neuropsychological studies, provides significant insights into early behavioral indicators of cognitive decline and predicts the onset of dementia in older adults. Although the importance of understanding mild behavioral impairment is acknowledged, comprehensive reviews of its correlates with older adults are limited.

**Objective:**

This scoping review aims to identify the impact of mild behavioral impairment on health outcomes in older adults and the factors associated with mild behavioral impairment.

**Methods:**

The review will adhere to the Joanna Briggs Institute’s methodological principles for scoping reviews. We will include studies focusing mainly on mild behavioral impairment in older adults, with the literature on this topic being limited to the period from 2003 to the present. Other clinical diagnoses, such as cognitive impairment, Parkinson disease, and multiple sclerosis, will not be included. We will use databases including PubMed (MEDLINE), CINAHL, Web of Science, Embase, PsycINFO, Cochrane, and Scopus for relevant articles published in English. Both gray literature and peer-reviewed articles will be considered during screening. Three independent reviewers will extract data using a predefined data extraction tool. Extracted data will be presented using tables, figures, and a narrative summary aligned with review questions, accompanied by an analysis of study characteristics and categorization of mild behavioral impairment correlates.

**Results:**

The results will be presented as a descriptive summary, structured according to the associated factors related to mild behavioral impairment, and the health outcomes. Additionally, the data on study characteristics will be presented in tabular format. An exploratory search was conducted in July 2023 to establish a comprehensive search strategy, and iterative refinements to the scoping review protocol and formalization of methods were completed. A follow-up search is planned for May 2024, with the aim of submitting the findings for publication in peer-reviewed journals.

**Conclusions:**

To our knowledge, this would be the first study to map the literature on the health-related factors and outcomes of mild behavioral impairment. The findings will support the development of interventions to prevent the occurrence of mild behavioral impairment and mitigate the negative outcomes of mild behavioral impairment.

**International Registered Report Identifier (IRRID):**

DERR1-10.2196/60009

## Introduction

As population aging continues to become an irreversible global trend, the prevalence of dementia is also expected to increase rapidly. The global prevalence of dementia in older adults is projected to double every 20 years, reaching 58 million in 2020, an anticipated 82 million in 2030, and 150 million in 2050 [1]. The financial cost of dementia was already substantial at US $ 818 billion in 2015 [2]. However, the recent COVID-19 pandemic has amplified the escalating costs of dementia care [3]. The economic impact of dementia extends beyond direct medical and social service expenses, such as treatment, medication, and social welfare, and encompasses indirect costs borne by caregivers and family members of older adults with dementia [4,5]. Caregivers often experience long-term stress, depression, anxiety, burnout, and physical health problems, which can limit their participation in economic activities, thereby amplifying the significant economic burden associated with dementia care [6,7]. The escalating health care burden presents a global social challenge that impacts not only the affected individuals but also families and communities, underscoring the urgent need for comprehensive strategies to address this pressing issue [8].

Although no cure for dementia currently exists, several studies have suggested that preventive interventions in the preclinical and prodromal stages can delay its onset and improve cognitive function by targeting modifiable risk factors [6,9]. The preclinical stage represents individuals who, despite not showing overt clinical signs of cognitive decline, have underlying dementia pathology. Meanwhile, those in the prodromal stage display evidence of clinical decline, demonstrated by subtle changes in cognition and short-term memory loss [10,11]. These stages have become focal points of recent research. Typically, the progression starts with normal cognition, transition into subjective cognitive decline (SCD)—a decrease in cognitive ability that is subjectively perceived compared to prior normal status but irrelevant to objective cognitive test results [12]—and eventually evolves into mild cognitive impairment (MCI), a condition characterized by a noticeable decline in cognitive abilities that does not interfere with daily functioning but increases the risk of Alzheimer disease or other types of dementia [13-15]. This transitional state of MCI poses a relatively high risk of progression to dementia, with some studies highlighting an estimated annual progression rate of approximately 10% [15-18]. However, not all cases of MCI inevitably progress to dementia [19]. The rate of return to normal cognition from MCI varies across studies, but one meta-analysis reports that approximately 24% of individuals with MCI revert to objectively normal cognition [20].

Recently, mild behavioral impairment (MBI), a neuropsychological syndrome that typically emerges later in life and can manifest across the entire predementia spectrum, has been introduced as a precursor to cognitive decline and dementia [21]. Prior to the development and implementation of preventive and supportive interventions for at-risk populations, it is essential to thoroughly understand MBI and its related factors that can influence multidimensional aspects of health in older adults.

According to the latest diagnostic guidelines published by the Alzheimer’s Association International Society to Advance Alzheimer’s Research and Treatment, MBI is characterized by behavioral and personality changes persisting for at least 6 months from the initial onset [22]. These changes, including reduced motivation, impaired emotional regulation, deficiency in impulse control, and inappropriate social behavior, minimally impact interpersonal relationships and workplace performance [23]. Evidence suggests that older adults with SCD who experience MBI are more likely to develop dementia [23]. Moreover, when MBI is accompanied by functional deterioration in daily life, it can serve as a neuropsychiatric symptom and prognostic marker of dementia [21,24]. Therefore, exploring the health outcomes of MBI has significant implications for dementia prevention and prediction of its progression.

Since the diagnostic criteria for MBI were first introduced in 2003 [25,26], MBI has been studied over the past two decades as a predictor and prognostic marker of cognitive impairment and dementia [23,27]. During this period, significant strides were made toward the conceptual understanding of MBI and the development of tailored diagnostic tools offering consistent and reliable means of assessing MBI, such as the Neuropsychiatric Inventory Questionnaire (NPI-Q) and the MBI Checklist [28-30]. The MBI Checklist was validated in comparison with the NPI-Q [31]. Furthermore, diverse methodologies have been employed in MBI research, including family interviews [32], neuropsychological tests [33], and imaging and cerebrospinal fluid diagnostic tests to identify MBI [34]. Several studies have also scrutinized differences in MBI manifestations according to sociodemographic characteristics [30,35] and have examined correlations between biomarkers [36], specific diseases, and MBI [37] as well as the impact of MBI on the progression from MCI to dementia [33,38].

However, as MBI is a relatively recent neurobehavioral concept, the literature lacks a comprehensive review addressing the correlates of MBI in older adults, apart from one meta-analysis on pathological and genetic relationships with MBI prevalence [39]. Given the potential insights into predicting and preventing dementia and assessing the risk of transition to this condition, a thorough review of the physiological, psychological, and social factors associated with the development and progression of MBI, as well as their influence on the relationship between MBI and other health outcomes, is crucial. Therefore, this scoping review aims to provide an overview of MBI and its correlates among older adults. Mapping evidence of factors correlated with MBI will guide the development of targeted preventative and supportive interventions for at-risk populations, laying the groundwork for interventions to promote preventive measures and improve self-management. This will also provide valuable insights for health care providers, enabling them to facilitate awareness and early detection of MBI. This, in turn, can lead to improved patient care and health outcomes. Furthermore, it underscores the importance of addressing behavioral symptoms later in life, and it advocates for targeted patient-centered clinical intervention and nursing care for older adults. The specific research questions are as follows:

1. What are the health outcomes associated with MBI in older adults?
2. What are the identified factors associated with MBI in older adults, and how have they been reported to be related to MBI?
3. What are the research trends in MBI, and what are the limitations and knowledge gaps in the existing studies?

## Methods

### Study Design

To identify the impact of MBI on health outcomes in older adults and factors associated with MBI, we seek to conduct a scoping review, which is the most suitable method for mapping evidence on this topic. This review follows the Joanna Briggs Institute’s methodology for scoping reviews [40] and reports the results according to the PRISMA-ScR (Preferred Reporting Items for Systematic Reviews and Meta-Analyses extension for Scoping Reviews) checklist [41]. An exploratory search took place in July 2023 to construct the full search strategy, yielding preliminary results. An update is scheduled for May 2024.

### Ethical Considerations

As this is a review of published and gray literature, ethical approval from a human research committee is not required.

### Search Methods

#### Sources of Evidence

This review will consider observational (including prospective and retrospective cohort studies, analytical cross-sectional studies, and case-control studies), experimental (including randomized and nonrandomized controlled trials), and qualitative studies for inclusion. We will include papers published in English from 2003 to date. Given that most previous research on MBI has been conducted in English, prioritizing English-language publications ensures consistency in terminology, which is critical for the search strategy and inclusion criteria and allows for interpreting and summarizing the impact of MBI on health outcomes accurately in accordance with international standards. Additionally, restricting the review to English-language publications minimizes the risk of variability and potential bias, allowing little impact on the overall integrity and conclusions of the review [42,43]. Books, editorials, commentaries, and letters will be excluded. However, we will include gray literature sources, such as reports, working papers, policy literature, government documents, and newsletters for a more comprehensive review.

#### Search Strategy

The search strategy will adhere to the PRISMA-ScR checklist [41]. Joanna Briggs Institute’s 3-step search strategy will be followed to ensure a comprehensive search. First, we will search MEDLINE and CINAHL to identify keywords and indexing terms in the titles and abstracts of potential articles that will be included in the review. This initial search will inform the development of the final search strategy. Second, these identified keywords and indexing terms will be used to search all the included databases. In other words, terms from the titles and abstracts of relevant articles, along with the indexing terms used to describe these articles, will be used to develop a comprehensive search strategy for multiple databases, specifically PubMed (MEDLINE), CINAHL, Web of Science, Embase, PsycINFO, Cochrane, and Scopus. After establishing a search formula using the appropriate subject words and consistent keywords in each database, the search formulae will be reviewed by a professional librarian at the university’s medical library. Tables S1–S7 in Multimedia Appendix 1 present the search strategy for each database. Third, after conducting the search using the previous strategies, we will analyze the bibliographies of the studies selected after the final search to identify relevant papers that may not be captured during the initial search. Our search strategy encompasses peer-reviewed published literature as well as gray literature, including doctoral dissertations to ensure comprehensive coverage.

#### Information Sources

The following databases will be searched: PubMed (MEDLINE), CINAHL, Web of Science, Embase, PsycINFO, Cochrane, and Scopus. During the search process, we may discover other potentially useful information sources and terms that will be incorporated into the review. To ensure completeness, we will contact the original authors of the studies to further clarify this information, if necessary. As scoping reviews primarily aim to map evidence rather than synthesize findings, we will not evaluate the methodological quality of the included sources of evidence.

### Inclusion and Exclusion Criteria

#### Population or Participants

This scoping review will include studies on older adults in the preclinical or prodromal stages of the dementia continuum, encompassing normal stages of SCD or MCI. Due to varying age thresholds for older adults in different studies, our review will adopt a broad approach, incorporating all studies that classify their population as “older adults” based on each study’s criteria. The term “older” refers to a specific group of adults who are chronologically older within the larger population of adults. The term has been broadly defined to include all studies that focus on recruiting older adults based on their criteria, such as being 60 years and older. Furthermore, the definition of older adults varies among countries, as diverse age standards exist for defining this population, according to the World Health Organization [44]. The diagnostic criteria for SCD and MCI adhere to the criteria established in each study.

Considering the challenges of identifying early SCD without solid empirical data, our search criteria will also encompass older adults who have not obtained a definitive diagnosis. This means including those diagnosed with normal cognition at the time of the study, essentially expanding our focus to include individuals on the threshold of cognitive decline. Studies on older individuals with other clinical diagnoses that can result in cognitive decline as one of its symptoms, such as dementia, Parkinson disease, or multiple sclerosis, will be excluded. However, to enhance the comprehensiveness of our review, we will include studies that conducted supplementary analyses, wherein patients with dementia were subsequently excluded, despite their initial inclusion within the subject groups.

#### Concept

MBI is the key concept in this review. We will include literature published from 2003 onwards, as the diagnostic criteria for MBI were first presented in 2003 in an academic journal [25,26].

#### Context

To thoroughly investigate the research questions related to MBI, there will be no restrictions on the study contexts or settings. A wide range of contexts will be considered eligible, including acute care, long-term care, and community-based environments.

### Study Selection

All identified citations will be collated and uploaded to EndNote 20 (Clarivate Analytics), and the duplicates will be removed following the search. The titles and abstracts will be screened by 3 independent reviewers to assess the inclusion criteria. Prior to embarking on source selection, pilot testing for the title and abstract screening will be undertaken to refine the inclusion/exclusion criteria and validate the reviewers’ screening process using a randomly selected sample of 50 titles and abstracts. Thereafter, the full texts of potentially relevant sources will be retrieved. The full texts of the selected articles will be assessed in detail against the inclusion criteria by 3 independent reviewers. This review will report the reasons for excluding sources that fail to meet the predetermined inclusion criteria. Any disagreements between the reviewers during any stage of the selection process will be resolved by involving an additional reviewer or through discussion. The final scoping review will include a comprehensive report on the search results and the process of study inclusion, along with a presentation of the findings using a flowchart based on PRISMA-ScR [41].

### Data Extraction

Three independent reviewers will utilize the data extraction tool, as presented in Multimedia Appendix 2. The extracted data will encompass specific participant details, concepts, contexts, study methods, and the main findings relevant to the review questions. The preliminary data extraction tool will be adjusted and refined throughout the data extraction process for each source, as required. These modifications will be documented in the final review. In the event of any discrepancies between the reviewers, we will resolve them through discussion or by consulting an additional reviewer. If necessary, the authors of the selected studies will be contacted to obtain any missing or supplementary data.

### Data Synthesis

The extracted data will be presented using tables and figures to address the review questions. Tables will be constructed to illustrate the classification of MBI correlates, with comprehensive descriptions of health outcomes (research question 1), related factors (research question 2), and critical assessment of research limitations (research question 3). Additionally, a narrative summary will discuss how the results correspond to the objectives and questions of the review. During the discussion of results, we will analyze the characteristics of the selected studies and categorize MBI correlates according to their subgroups. The methods for presenting the results may be further refined during the review process, with details provided in the final review.

## Results

An exploratory search was conducted in July 2023, and a comprehensive search strategy was formulated based on the preliminary results. We reviewed studies on MCI and SCD in older adults in the exploratory search and recognized the difficulty in detecting early SCD without substantial empirical data in individuals diagnosed with normal cognition at the study’s inception. Therefore, as noted in the *Methods* section, we broadened our review scope to include studies of older adults across the spectrum from normal cognition to MCI, refining our search strategy with a librarian's assistance. Iterative refinements to the scoping review protocol and formalization of methods were completed. A subsequent search is scheduled for May 2024, with the objective of submitting the results for publication in peer-reviewed journals.

## Discussion

### Expected Findings

This study introduces a protocol outlining a scoping review designed to address specific research questions regarding the correlates of MBI. The main findings of the review will identify key physiological, psychological, and social factors associated with MBI in older adults. Previous research has largely focused on the prevalence or individual domains of MBI, but this review will fill a significant gap in the literature, providing a broader understanding of how various factors contribute to MBI and the diverse ways MBI can impact health outcomes; to the best of our knowledge, such a review has not yet been conducted.

### Strengths and Limitations

Our review will provide a timely and valuable contribution, encompassing evidence from various fields and methodological approaches and focusing on the physiological, psychological, and social factors associated with MBI in older adults. Understanding these factors and their influence on the relationship between MBI and health outcomes is crucial for proposing preventive measures and improving self-management strategies for person-centered intervention. This review will also contribute to the development of targeted preventive and supportive interventions aimed at alleviating the influence of MBI on health outcomes in older adults. Since this review will comprehensively map the correlates of MBI rather than measuring effectiveness, it will not perform a quality appraisal. Additionally, since non-English literature will not be included, some relevant studies may be missed which will reduce the generalizability of the findings. However, to increase the identification of eligible studies, we selected seven databases, including databases for gray literature, and developed appropriate search strategies.

### Conclusions

This study will provide significant insights into the health-related factors and outcomes of MBI. Finally, it will lay the groundwork for policy development, fostering a broader social understanding of MBI, which can be a crucial focal point for future interventions targeting the at-risk group for dementia. Our knowledge dissemination strategy will employ various approaches, including peer-reviewed journals, webinars, and conference presentations. Collaborating with national research networks will extend our reach and inform policy, practice, and research. Recognizing the necessity for detailed dissemination plans, we will enhance our strategy by outlining specific channels to share our findings with academic communities, policy makers, and practitioners.

## Appendix

Multimedia Appendix 1 Search strategy.

Multimedia Appendix 2 Data extraction instrument.

## Abbreviations

MBI: mild behavioral impairment
MCI: mild cognitive impairment
NPI-Q: Neuropsychiatric Inventory Questionnaire
PRISMA-ScR: Preferred Reporting Items for Systematic Reviews and Meta-Analyses extension for Scoping Reviews
SCD: subjective cognitive decline
