# Supplementary material for: Correlates of Mild Behavioral Impairment in Older Adults: Protocol for a Scoping Review
Source: JMIR Res Protoc. 2024 Jul 29;13:e60009. doi: 10.2196/60009 (PMC11319883; doi:10.2196/60009)
Supplement: Multimedia Appendix 1 [file resprot_v13i1e60009_app1.docx]

**Appendix 1**

## **Supplementary Table 1.** PubMed Search Strategy

| Search # | MeSH Terms and Keywords |
| --- | --- |
| #1 | "Old Age"[Title/Abstract] OR "Old People"[Title/Abstract] OR "Older"[Title/Abstract] OR "Older Adult*"[Title/Abstract] OR "Older People"[Title/Abstract] OR "Elderly"[Title/Abstract] OR "Elderly Adult*"[Title/Abstract] OR "Elder"[Title/Abstract] OR "Senior"[Title/Abstract] OR "Later Life"[Title/Abstract] OR "Aged"[Title/Abstract] Or "Geriatrics"[Title/Abstract] OR "Aged"[MeSH Terms] OR "Geriatrics"[MeSH Terms] |
| #2 | "Mild Behavioral Impairment"[Title/Abstract] OR "Neuropsychiatric Symptoms"[Title/Abstract] OR "Non-cognitive symptom*"[Title/Abstract] |
| #3 | "Without Dementia"[Title/Abstract] OR "Pre-Clinical Dementia"[Title/Abstract] OR "Preclinical Dementia"[Title/Abstract] OR "Pre-Dementia"[Title/Abstract] OR "Predementia"[Title/Abstract] OR "Prodromal Dementia"[Title/Abstract] OR "Cognitive Impairment"[Title/Abstract] OR "Cognitive Decline"[Title/Abstract] OR "Mild Cognitive Impairment"[Title/Abstract] OR "Subjective Cognitive Decline"[Title/Abstract] OR "Subjective Cognitive Impairment"[Title/Abstract] OR "Self-reported Cognitive Decline"[Title/Abstract] OR "Subjective Cognitive Complain*"[Title/Abstract] OR "Self-reported Cognitive Complain*"[Title/Abstract] OR "Self-reported Cognitive Impairment"[Title/Abstract] OR "Subjective Memory Complain*"[Title/Abstract] OR "Self-reported Memory Complain*"[Title/Abstract] OR "Subjective Memory Impairment"[Title/Abstract] OR "Self-reported Memory Impairment"[Title/Abstract] OR "Subjective Memory Decline"[Title/Abstract] OR "Self-reported Memory Decline"[Title/Abstract] OR "Subjective Memory Loss"[Title/Abstract] OR "Cognitively Normal"[Title/Abstract] OR "Normal Cognition"[Title/Abstract] OR "Cognitive Dysfunction"[Title/Abstract] OR "Cognitive Defect"[Title/Abstract] OR "Neurocognitive Disorders"[Title/Abstract] OR "Prodrome"[Title/Abstract] OR "Prodromal Symptoms"[Title/Abstract] OR "Cognitive Disorders"[Title/Abstract] OR "Cognition Disorders"[Title/Abstract] OR "Memory Decline"[Title/Abstract] OR "Memory Disorders"[Title/Abstract] OR "Cognitive Dysfunction"[Mesh:NoExp] OR "Prodromal Symptoms"[MeSH Terms] OR "Memory Disorders"[Mesh:NoExp] |

## **Supplementary Table 2.** CINAHL Search Strategy

| Search # | Subject Headings (MH) and Keywords |
| --- | --- |
| S1 | TI("Old Age" OR "Old People" OR "Older" OR "Older Adult*" OR "Older People" OR "Elderly" OR "Elderly Adult*" OR "Elder" OR "Senior" OR "Later Life" OR "Aged" OR "Geriatrics") OR AB("Old Age" OR "Old People" OR "Older" OR "Older Adult*" OR "Older People" OR "Elderly" OR "Elderly Adult*" OR "Elder" OR "Senior" OR "Later Life" OR "Aged" OR "Geriatrics") OR (MH "Aged+") OR (MH "Geriatrics") |
| S2 | TI("Mild Behavioral Impairment" OR "Neuropsychiatric Symptoms" OR "Non-cognitive symptom*") OR AB("Mild Behavioral Impairment" OR "Neuropsychiatric Symptoms" OR "Non-cognitive symptom*") |
| S3 | TI("Without Dementia" OR "Pre-Clinical Dementia" OR "Preclinical Dementia" OR "Pre-Dementia" OR "Predementia" OR "Prodromal Dementia" OR "Cognitive Impairment" OR "Cognitive Decline" OR "Mild Cognitive Impairment" OR "Subjective Cognitive Decline" OR "Subjective Cognitive Impairment" OR "Self-reported Cognitive Decline" OR "Subjective Cognitive Complain*" OR "Self-reported Cognitive Complain*" OR "Self-reported Cognitive Impairment" OR "Subjective Memory Complain*" OR "Self-reported Memory Complain*" OR "Subjective Memory Impairment" OR "Self-reported Memory Impairment" OR "Subjective Memory Decline" OR "Self-reported Memory Decline" OR "Subjective Memory Loss" OR "Cognitively Normal" OR "Normal Cognition" OR "Cognitive Defect" OR "Cognitive Dysfunction" OR "Neurocognitive Disorders" OR "Prodrome" OR "Prodromal Symptoms" OR "Memory Disorders" OR "Cogniti* Disoders" OR "Memory Decline") OR AB("Without Dementia" OR "Pre-Clinical Dementia" OR "Preclinical Dementia" OR "Pre-Dementia" OR "Predementia" OR "Prodromal Dementia" OR "Cognitive Impairment" OR "Cognitive Decline" OR "Mild Cognitive Impairment" OR "Subjective Cognitive Decline" OR "Subjective Cognitive Impairment" OR "Self-reported Cognitive Decline" OR "Subjective Cognitive Complain*" OR "Self-reported Cognitive Complain*" OR "Self-reported Cognitive Impairment" OR "Subjective Memory Complain*" OR "Self-reported Memory Complain*" OR "Subjective Memory Impairment" OR "Self-reported Memory Impairment" OR "Subjective Memory Decline" OR "Self-reported Memory Decline" OR "Subjective Memory Loss" OR "Cognitively Normal" OR "Normal Cognition" OR "Cognitive Defect" OR "Cognitive Dysfunction" OR "Neurocognitive Disorders" OR "Prodrome" OR "Prodromal Symptoms" OR "Memory Disorders" OR "Cogniti* Disoders" OR "Memory Decline") OR (MM "Mild Cognitive Impairment") OR (MH "Cognition Disorder") OR (MH "Memory Disorders") |

## **Supplementary Table 3.** CINAHL Search Strategy

| Search # | Main Subjects and Keywords |
| --- | --- |
| S1 | TIAB("Old Age" OR "Old People" OR "Older" OR "Older Adult*" OR "Older People" OR "Elderly" OR "Elderly Adult*" OR "Elder" OR "Senior" OR "Later Life" OR "Aged" OR "Geriatrics") OR MAINSUBJECT.EXACT("Older Adulthood") OR MAINSUBJECT.EXACT.EXPLODE("Geriatrics") |
| S2 | TIAB("Mild Behavioral Impairment" OR "Neuropsychiatric Symptoms" OR "Non-cognitive symptom*") |
| S3 | TIAB("Without Dementia" OR "Pre-Clinical Dementia" OR "Preclinical Dementia" OR "Pre-Dementia" OR "Predementia" OR "Prodromal Dementia" OR "Cognitive Impairment" OR "Cognitive Decline" OR "Mild Cognitive Impairment" OR "Subjective Cognitive Decline" OR "Subjective Cognitive Impairment" OR "Self-reported Cognitive Decline" OR "Subjective Cognitive Complain*" OR "Self-reported Cognitive Complain*" OR "Self-reported Cognitive Impairment" OR "Subjective Memory Complain*" OR "Self-reported Memory Complain*" OR "Subjective Memory Impairment" OR "Self-reported Memory Impairment" OR "Subjective Memory Decline" OR "Self-reported Memory Decline" OR "Subjective Memory Loss" OR "Cognitively Normal" OR "Normal Cognition" OR "Cognitive Dysfunction" OR "Cognitive Defect" OR "Neurocognitive Disorders" OR "Prodrome" OR "Prodromal Symptoms" OR "Memory Disorders" OR "Cogniti* Disorders" OR "Memory Decline") OR MAINSUBJECT.EXACT("Neurocognitive Disorders") OR MAINSUBJECT.EXACT("Mild Cognitive Impairment") OR MAINSUBJECT.EXACT("Prodrome") OR MAINSUBJECT.EXACT("Cognitive Impairment") OR MAINSUBJECT.EXACT("Memory Disorders") |

## **Supplementary Table 4.** Embase Search Strategy

| Search # | MeSH Terms and Keywords |
| --- | --- |
| #1 | ('old age':ti,ab,kw OR 'old people':ti,ab,kw OR 'older':ti,ab,kw OR 'older adult*':ti,ab,kw OR 'older people':ti,ab,kw OR 'elderly':ti,ab,kw OR 'elderly adult*':ti,ab,kw OR 'elder':ti,ab,kw OR 'senior':ti,ab,kw OR 'later life':ti,ab,kw OR 'aged':ti,ab,kw OR 'geriatrics':ti,ab,kw OR 'aged'/exp OR 'geriatrics'/exp) |
| #2 | ('mild behavioral impairment':ti,ab,kw OR 'neuropsychiatric symptoms':ti,ab,kw OR 'non-cognitive symptom*':ti,ab,kw) |
| #3 | ('without dementia':ti,ab,kw OR 'pre-clinical dementia':ti,ab,kw OR 'preclinical dementia':ti,ab,kw OR 'pre-dementia':ti,ab,kw OR 'predementia':ti,ab,kw OR 'prodromal dementia':ti,ab,kw OR 'cognitive impairment':ti,ab,kw OR 'cognitive decline':ti,ab,kw OR 'mild cognitive impairment':ti,ab,kw OR 'subjective cognitive decline':ti,ab,kw OR 'subjective cognitive impairment':ti,ab,kw OR 'self-reported cognitive decline':ti,ab,kw OR 'subjective cognitive complain*':ti,ab,kw OR 'self-reported cognitive complain*':ti,ab,kw OR 'self-reported cognitive impairment':ti,ab,kw OR 'subjective memory complain*':ti,ab,kw OR 'self-reported memory complain*':ti,ab,kw OR 'subjective memory impairment':ti,ab,kw OR 'self-reported memory impairment':ti,ab,kw OR 'subjective memory decline':ti,ab,kw OR 'self-reported memory decline':ti,ab,kw OR 'subjective memory loss':ti,ab,kw OR 'cognitively normal':ti,ab,kw OR 'normal cognition':ti,ab,kw OR 'cognitive dysfunction':ti,ab,kw OR 'cogniti* disorders':ti,ab,kw OR 'cognitive defect':ti,ab,kw OR 'neurocognitive disorders':ti,ab,kw OR 'memory decline' OR 'prodrome':ti,ab,kw OR 'prodromal symptoms':ti,ab,kw OR 'memory disorders':ti,ab,kw OR 'mild cognitive impairment'/exp OR 'cognitive defect'/de OR 'prodromal symptom'/exp OR 'memory disorder'/de) |

## **Supplementary Table 5.** Cochrane Library Search Strategy

| Search # | MeSH Terms and Keywords |
| --- | --- |
| #1 | ("Aged" OR “Geriatrics” OR “old age” OR “old people” OR “older” OR “older people” OR “elderly” OR “elderly adult” OR “elder” OR “senior”):ti OR ("Aged" OR “Geriatrics” OR “old age” OR “old people” OR “older” OR “older people” OR “elderly” OR “elderly adult” OR “elder” OR “senior”):ti,ab,kw |
| #2 | MeSH descriptor: [Aged] explode all trees |
| #3 | MeSH descriptor: [Geriatrics] explode all trees |
| #4 | ("Mild Behavioral Impairment" OR "Neuropsychiatric Symptoms" OR "Non-cognitive symptom"):ti OR ("Mild Behavioral Impairment" OR "Neuropsychiatric Symptoms" OR "Non-cognitive symptom"):ti,ab,kw |
| #5 | ("Without Dementia" OR "Pre-Clinical Dementia" OR "Preclinical Dementia" OR "Pre-Dementia" OR "Predementia" OR "Prodromal Dementia" OR "Cognitive Impairment" OR "Cognitive Decline" OR "Mild Cognitive Impairment" OR "Subjective Cognitive Decline" OR "Subjective Cognitive Impairment" OR "Self-reported Cognitive Decline" OR "Subjective Cognitive Complaint" OR "Subjective Cognitive Complaints" OR "Self-reported Cognitive Complaint" OR "Self-reported Cognitive Complaints" OR "Self-reported Cognitive Impairment" OR "Subjective Memory Complaint" OR "Subjective Memory Complaints" OR "Self-reported Memory Complaint" OR "Self-reported Memory Complaints" OR "Subjective Memory Impairment" OR "Self-reported Memory Impairment" OR "Subjective Memory Decline" OR "Self-reported Memory Decline" OR "Subjective Memory Loss" OR "Cognitively Normal" OR "Normal Cognition" OR "Cognitive Dysfunction" OR "Cognitive Defect" OR "Cognition Disorders" OR "Cognitive Disorders" OR "Neurocognitive Disorders" OR "Prodrome" OR "Prodromal Symptoms" OR “Memory Decline” OR "Memory Disorders"):ti,ab,kw |
| #6 | MeSH descriptor: [Cognitive Dysfunction] this term only |
| #7 | MeSH descriptor: [Prodromal Symptoms] explode all trees |
| #8 | MeSH descriptor: [Memory Disorders] this term only |

## **Supplementary Table 6.** Scopus Search Strategy

| Search # | MeSH Terms and Keywords |
| --- | --- |
| #1 | TITLE-ABS-KEY ( "Old Age" OR "Old People" OR "Older" OR "Older Adult*" OR "Older People" OR "Elderly" OR "Elderly Adult*" OR "Elder" OR "Senior" OR "Later Life" OR "Aged" OR "Geriatrics" ) |
| #2 | TITLE-ABS-KEY ( "Mild Behavioral Impairment" OR "Neuropsychiatric Symptoms" OR "Non-cognitive symptom*" ) |
| #3 | TITLE-ABS-KEY ( "Without Dementia" OR "Pre-Clinical Dementia" OR "Preclinical Dementia" OR "Pre-Dementia" OR "Predementia" OR "Prodromal Dementia" OR "Cognitive Impairment" OR "Cognitive Decline" OR "Mild Cognitive Impairment" OR "Subjective Cognitive Decline" OR "Subjective Cognitive Impairment" OR "Self-reported Cognitive Decline" OR "Subjective Cognitive Complain*" OR "Self-reported Cognitive Complain*" OR "Self-reported Cognitive Impairment" OR "Subjective Memory Complain*" OR "Self-reported Memory Complain*" OR "Subjective Memory Impairment" OR "Self-reported Memory Impairment" OR "Subjective Memory Decline" OR "Self-reported Memory Decline" OR "Subjective Memory Loss" OR "Cognitively Normal" OR "Normal Cognition" OR "Cognitive Dysfunction" OR "cogniti* disorders" OR "Cognitive Defect" OR "Neurocognitive Disorders" OR "Prodrome" OR "Prodromal Symptoms" OR "Memory Disorder" OR “Memory Decline” ) |

## **Supplementary Table 7.** Web of Science Search Strategy

| Search # | MeSH Terms and Keywords |
| --- | --- |
| #1 | TS=("Old Age" OR "Old People" OR "Older" OR "Older Adult*" OR "Older People" OR "Elderly" OR "Elderly Adult*" OR "Elder" OR "Senior" OR "Later Life" OR "Aged" OR "Geriatrics") |
| #2 | TS=("Mild Behavioral Impairment" OR "Neuropsychiatric Symptoms" OR "Non-cognitive symptom*") |
| #3 | TS=("Without Dementia" OR "Pre-Clinical Dementia" OR "Preclinical Dementia" OR "Pre-Dementia" OR "Predementia" OR "Prodromal Dementia" OR "Cognitive Impairment" OR "Cognitive Decline" OR "Mild Cognitive Impairment" OR "Subjective Cognitive Decline" OR "Subjective Cognitive Impairment" OR "Self-reported Cognitive Decline" OR "Subjective Cognitive Complain*" OR "Self-reported Cognitive Complain*" OR "Self-reported Cognitive Impairment" OR "Subjective Memory Complain*" OR "Self-reported Memory Complain*" OR "Subjective Memory Impairment" OR "Self-reported Memory Impairment" OR "Subjective Memory Decline" OR "Self-reported Memory Decline" OR "Subjective Memory Loss" OR "Cognitively Normal" OR "Normal Cognition" OR "Cognitive Dysfunction" OR "Cognitive Defect" OR "Neurocognitive Disorders" OR "Prodrome" OR "Prodromal Symptoms" OR "Memory Disorders" OR "Cogniti* Disorders" OR "Memory Decline") |
