# Supplementary material for: Correlates of Mild Behavioral Impairment in Older Adults: Protocol for a Scoping Review
Source: JMIR Res Protoc. 2024 Jul 29;13:e60009. doi: 10.2196/60009 (PMC11319883; doi:10.2196/60009)
Supplement: Multimedia Appendix 2 [file resprot_v13i1e60009_app2.docx]

**Appendix 2**

| Author | Year | Title | Type of publication | Study design | Methods | Aim | Setting/  Country | Study participants | Type of cognitive impairment | IV | DV | MBI diagnosis | Main Outcome | Additional findings /Comments |
| --- | --- | --- | --- | --- | --- | --- | --- | --- | --- | --- | --- | --- | --- | --- |
|  |  |  |  |  |  |  |  |  |  |  |  |  |  |  |
|  |  |  |  |  |  |  |  |  |  |  |  |  |  |  |
|  |  |  |  |  |  |  |  |  |  |  |  |  |  |  |
|  |  |  |  |  |  |  |  |  |  |  |  |  |  |  |
|  |  |  |  |  |  |  |  |  |  |  |  |  |  |  |
|  |  |  |  |  |  |  |  |  |  |  |  |  |  |  |
|  |  |  |  |  |  |  |  |  |  |  |  |  |  |  |
|  |  |  |  |  |  |  |  |  |  |  |  |  |  |  |
|  |  |  |  |  |  |  |  |  |  |  |  |  |  |  |

Data extraction instrument

^*^ IV= Instrumental Variable, DV=Dependent Variable, MBI=Mild Behavioral Impairment
